# Design and Optimization of Colorimetric Paper-Based Analytical Device for Rapid Detection of Allopurinol in Herbal Medicine

**DOI:** 10.1155/2019/4682839

**Published:** 2019-03-19

**Authors:** Rimadani Pratiwi, Rieda Nurwulan Septyani, Rossi Febriany, Febrina Amelia Saputri, Rina Fajri Nuwarda

**Affiliations:** Department of Pharmaceutical Analysis and Medicinal Chemistry, Faculty of Pharmacy, Universitas Padjadjaran, Jatinangor, 45363, Indonesia

## Abstract

Traditional herbal medicine in Indonesia is still in great demand and popular in society. The Indonesian government regulations state that herbal medicine should not contain chemical drug due to the toxic effect of uncontrolled consumption. Allopurinol is one of the drugs commonly added to herbal medicine for the treatment of chronic gout. Paper-based analytical device is one of the latest forms of analysis that has been widely used for the identification of chemical elements, environmental contamination, bacteria, and many more. In this study, experiments were conducted using Whatman filter paper No. 1, No. 2, and No. 4 and Whatman chromatography as a paper, and 9 colorimetric reagents were tested for allopurinol detection in herbal medicine. There were 5 specific reagents that reacted positively with allopurinol and only 3 reagents that can be applied to the paper, that is, Folin-Ciocalteu, Tollens, and p-DAB reagent. The results of the optimization show that the most optimal immersion time was 60 minutes with a drying time of 30 minutes at 50°C. Each filter paper has different characteristic; however, there was no significant difference when all of the papers were used as PAD for allopurinol detection.

## 1. Introduction

Traditional herbal medicines contain herb combining or plant extracts to maintain good health and treat diseases [1]. Since the use of traditional medicine increases in Indonesian society, chemical drugs have been added to some of the traditional herbal medicine to enhance the therapeutic effect. One of them is allopurinol which is added to the herbal medicine for gout treatment. Meanwhile, the Indonesian Ministry of Health regulations state that herbal medicine should not contain synthetic chemicals or medicinal isolation results [2]. Allopurinol is a xanthine oxidase inhibitor drug for lowering blood uric acid levels frequently used for the treatment of chronic gout [3]. Chronic gout is signed by crystal formation in the joint caused by high levels of uric acid in the blood [4]. Allopurinol will inhibit xanthine oxidase enzyme to produce uric acid. Long-term consumption of allopurinol will cause several effects, especially in uncontrolled consumption. In hospitalized patients it has been reported that allopurinol shows acute adverse reaction like hematological abnormalities, diarrhea, and drug fever [5]. Therefore, monitoring allopurinol in herbal medicine is important to control the potential toxicity of allopurinol.

Several analytical methods such as spectrophotometric determination [6] and high-performance liquid chromatography (HPLC) [7] are used for allopurinol detection. Since both methods are selective and sensitive, they require expensive instrument and cannot be used for on-site analysis. Development of indicator strip or optical sensor membrane for detection chemical drug in herbal medicine is growing. Some researchers use the polymer as a substrate material [2, 8]. Recently, the paper-based analytical device (PAD) has attracted considerable attention because it is a simple, low-cost, and easy-to-use tool for environmental and biological analysis [9–11]. PAD has become effective in the on-site analysis for organic molecules [12], metals [9], and pesticides [13]. One of the most common detection methods used in PAD is a colorimetric method using specific reagent to identify and detect an analyte in a sample [14–16].

In this work, nine colorimetric reagents were screened to find the best colorimetric reagent for allopurinol detection. These nine colorimetric reagents were chosen based on the reaction between the functional group on allopurinol and a general reagent that was used as a colorimetric reagent. They are Dragendorff reagent, ferric chloride, Folin-Ciocalteu reagent, sodium nitroprusside, p-DAB reagent, Schiff reagent, potassium chlorate, Tollens reagent, and sodium nitrite [17, 18]. The Dragendorff reagent, p-DAB reagent, and diazotisation reaction were used to detect amines group in allopurinol. Ferric chloride was used to analyze a phenolic compound, fatty acid, or a phenylpyrazole. Folin-Ciocalteu was used to analyze phenolic compound and sodium nitroprusside for ketones or acetaldehyde. Tollens and Schiff reagent were used to detect aldehyde or ketone group. Potassium chlorate was used in amalic acid test; this test is specifically used to detect xanthine group in the compound. These reagents were screened and applied to the various Whatman filter papers, including Whatman No. 1, No. 4, and No. 6 and Whatman 1 chromatography. The selectivity, sensitivity, and application of this PAD to herbal medicine were also investigated. The result shows that this PAD is applicable to allopurinol detection in herbal medicine.

## 2. Material and Methods

All chemicals used were of analytical grade, and they were used without further purification. All solutions were made using distilled water. Allopurinol was obtained from Nanjing Pharma Chemical Plant. 2-Naphthol, ammonium hydroxide 25%, hydrochloric acid 37%, bismuth subnitrate, ethanol 95%, ferric chloride, potassium hydroxide, potassium iodide, sodium hydroxide, potassium chlorate crystals, sodium nitrite, sodium nitroprusside, sodium sulfite anhydrous, silver nitrate, Dragendorff reagent, p-DAB, and Folin-Ciocalteu reagent were purchased from Merck. Alkaline fuchsin was purchased from Sigma Aldrich, and sample packaged herbs were purchased from traditional drug store around Jatinangor, Sumedang, West Java. Whatman No. 1, No. 4, and No. 6 and Whatman 1 chromatography qualitative-grade filter paper were purchased from GE Healthcare. The absorbance measurement was recorded by UV-Visible spectrophotometer Analytik Jena SPECORD 200 using a 1.0 cm quartz cell.

### 2.1. Reagent Preparation

Nine reagents were used in this experiment. Dragendorff, p-DAB, and Folin-Ciocalteu reagent were directly used from Merck. Ferric chloride 5% was made in water. Sodium nitroprusside 1% was made in water. Schiff reagent was made by dissolving 200 mg of base fuchsin in 120 mL of hot water and letting it cool. Then, we added 2 g of sodium sulfite anhydrous in 20 mL of water, added 2 mL of concentrated HCl, diluted with water up to 200 mL, and left it for at least 2 hours. the p-DAB reagent was made by dissolving 2 g of p-DAB into 50 mL ethanol 95% and 50 mL concentrated HCl; reagents must be made fresh. Amalic acid test was performed using HCl 10 M, potassium chlorate, and ammonium hydroxide 2 M as a reagent. Tollens reagent was made by dissolving silver nitrate in 100 mL of aquadest, and 5 mL of NaOH 5% was added. After that 7.5 mL of concentrated ammonium hydroxide was added. Diazotization test was carried out by dissolving the sample in HCl 2 M, adding 1 drop sodium nitrite 1%, and adding 1 drop of 2-naft-2-ol 4% in NaOH 2 M. These reagents were then tested against allopurinol solution (100 mg/ml) and NaOH 2 N as a blank reagent to observe the color changes.

### 2.2. Design and Optimization of Specific Reagent Tests on Paper

**Figure 1:**
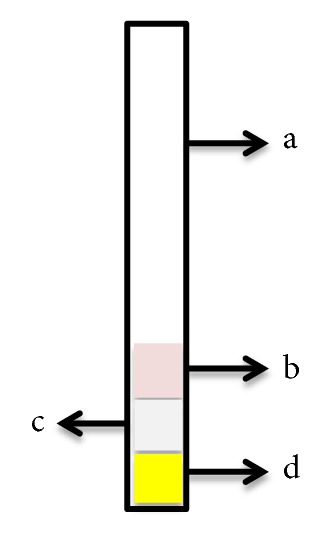
Design of paper-based analytical device: (a) support base, (b) Whatman filter paper content reagent 1, (c) Whatman filter paper content reagent 2, (d) Whatman filter paper content reagent 3.

Whatman filter paper No. 1, No. 4, and No. 6 and Whatman 1 chromatography were cut into 1 × 1 cm pieces, soaked in allopurinol specific reagents at 10, 20, 40, and 60 minute intervals, and then dried at room temperature and oven at 50°C. PAD that shows the fastest color change between all the time intervals was used for further research. The paper-based analytical device was designed following the design of a universal pH indicator strip as shown in Figure 1. The design consisted of photo paper as a support base (5 x 0.5 cm) and Whatman filter paper (1 x 1 cm) as a content specific reagent. Whatman filter paper that has been soaked with specific reagents is attached to the support base using double tape. The performance of PAD was observed by sensitivity test and selectivity test.

### 2.3. Application of PAD for Real Sample Analysis

To demonstrate the applicability of PAD for real sample analysis, the herbal medicine with or without registration number was analyzed using Thin Layer Chromatography (TLC), Spectrophotometry UV-Visible, and PAD. A total of 400 mg of herbal medicine has been finely powdered, dissolved with 10 ml 0.1 N NaOH, then sonicated for 15 minutes, and filtered. 1 mL of each herbal solution was diluted to 10 mL with 0.1 NaOH. For TLC analysis, the silica gel GF 254 was used as stationary phase and the solution mixture of 5 mL n-butanol: 5 mL 25% ammonium hydroxide was used as mobile phase. Paracetamol and allopurinol were used as the standard compound. The analysis was observed at 254 nm UV lamp. For Spectrophotometry UV-Visible analysis, 500 mg of herbal medicine that has been finely powdered and dissolved with 0.1 N NaOH as much as 10 mL, was sonicated for 15 minutes and filtered. Then the solution was added with 0.1 M HCl to 100 mL. 10 mL of the solution was diluted to 100 mL with 0.1 M HCl. Then the solution was diluted again to 10 mL and then 100 mL HCl 0.1 M was added. The absorbance spectra were recorded at 200-300 nm. For PAD analysis, 400 mg herbal medicine sample was dissolved in 10 mL of distilled water and 10 mL 2 M NaOH and then filtered. The PAD was immersed in the sample and the color change that occurs was observed.

## 3. Result and Discussion

### 3.1. Characterization of the Interaction between Colorimetric Reagent and Allopurinol in Solution

**Figure 2:**
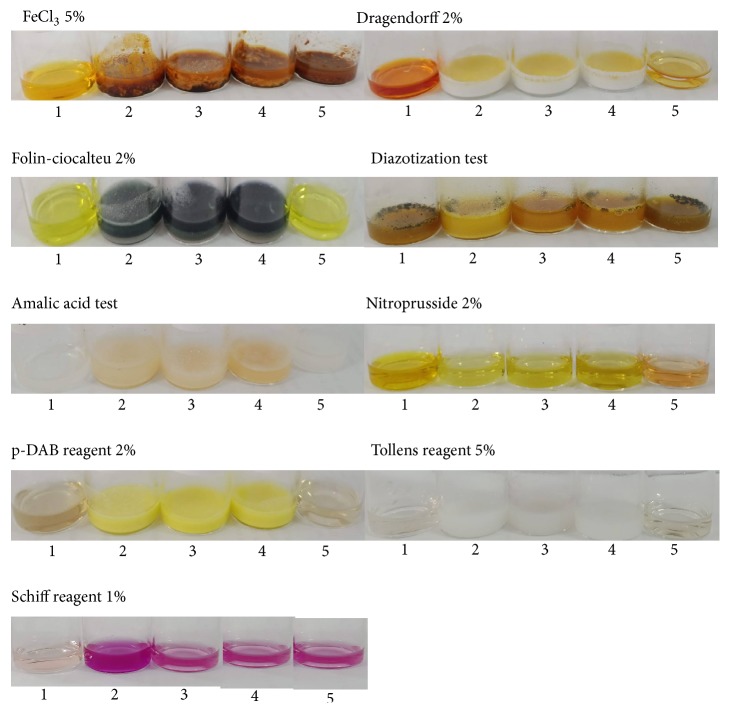
Color change of colorimetric reagent in the presence of (1) reagent, (2-4) allopurinol solution (100 mg/ml), and (5) NaOH 2 N as a blank reagent.

The colorimetric sensing ability of 9 reagents was tested against the allopurinol standard. They are Dragendorff reagent, ferric chloride, Folin-Ciocalteu reagent, sodium nitroprusside, p-DAB reagent, Schiff reagent, potassium chlorate, Tollens reagent, and sodium nitrite.  Figure 2 shows the color change of colorimetric reagent in the presence of allopurinol solution and NaOH 2 N as a blank reagent.

Figure 2 shows that some of the colors of the reagents changed after allopurinol solution was added to the reagent solution. Folin-Ciocalteu gives a dark blue color when reacting with allopurinol solution [18] while the color of FeCl_3_ reagent did not change after allopurinol was added. Diazotization test gives a dark red or orange-red colors when reacting with the amine group in allopurinol compound. However, the color change is not significant with the color of reagent, so this reagent was no longer used. Dragendorff reagent gives an orange, red-orange, or brown-orange deposits when reacting with amino group in the compound. The results show that, when this reagent reacts with allopurinol, the color became two-layer and cannot continue in the paper. p-DAB reagent gives a yellow color when reacting with an amine group in the compound. Nitroprusside reagent also did not continue because the color of reagent did not change. Schiff reagent gives a purple or violet color in the presence of aldehyde or ketone in the compound. Shift reagent cannot continue because the color change was caused by the basic condition from NaOH as blank reagent not by allopurinol compound. Tollens gives a silver precipitate when the reagent reacts with a carbonyl group on allopurinol solution. The amalic acid test gives a red, pink, or violet color when the reagent reacts with xanthine on allopurinol solution after the heating process. In this experiment, the heating procedure is avoided, because it is not applicable for PAD. Based on the result, Folin-Ciocalteu, p-DAB, and Tollens reagents continue to be used in PAD.

### 3.2. Design and Optimization of Specific Reagent Tests on Paper

**Figure 3:**
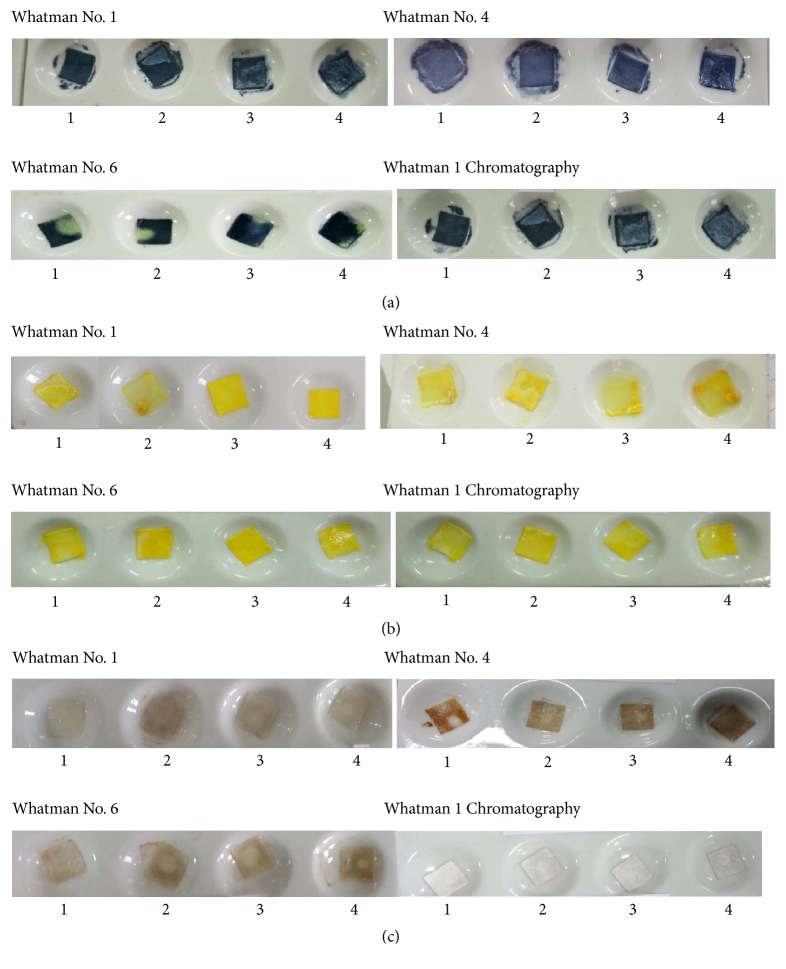
Color change of Whatman filter paper in the presence of allopurinol in a variation of immersion time: (1) 10 min; (2) 20 min; (3) 40 min; (4) 60 min. (a) Folin-Ciocalteu reagent + allopurinol; (b) p-DAB reagent + allopurinol; (c) Tollens reagent + allopurinol.

The Whatman filter paper used in this research was Whatman filter paper No. 1, No. 4, and No. 6 and Whatman 1 chromatography. Whatman No. 1 filter paper is the most widely used paper for routine analysis with moderate retention and flow rates with a pore size of 11*µ*m. Whatman No. 4 is filter paper with a very fast filtration speed with pore size 20-25*µ*m. Whatman No. 6 has a pore size of 3 *µ*m and was applied for water analysis, whereas Whatman 1 chromatography is the most widely used paper for chromatography analysis [19].  Figure 3 shows the color change of Whatman filter paper in the presence of allopurinol in a variation of immersion time.

**Figure 4:**
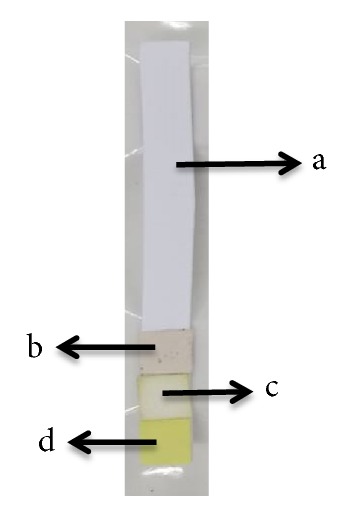
Design of paper-based analytical device: (a) support base, (b) Whatman filter paper content Folin-Ciocalteu reagent, (c) Whatman filter paper content p-DAB reagent, (d) Whatman filter paper content Tollens reagent.

The result shows the optimal immersion time is 60 minutes because at this time the color intensity is more intense than the other time. Each Whatman filter paper has different characteristic, especially the pore size. The smaller pore size will absorb more reagent. However, all papers do not have a significant difference in absorbing reagents and react with allopurinol. The optimum drying time was carried out at room temperature and in the oven. At room temperature, the time needed for the paper to dry completely is approximately 1 hour, whereas at a temperature of 50°C in the oven, the time needed for the paper to dry completely is 30 minutes. So the temperature and drying time of the paper used are set at 50°C for 30 minutes. The design of the paper-based analytical device is shown in Figure 4. The material used in this design is simple and cheap. This design was made for each filter paper.

### 3.3. Performance of Paper-Based Analytical Device

The sensitivity test of each filter paper was carried out by determining the lowest measurable concentration of allopurinol that can be detected by PAD. The different amount of allopurinol (0-100 mg/ml) was added to the PAD. The color change of PAD in the presence of allopurinol was observed. The color changes on each paper occur in a short time when it is immersed in allopurinol solution. All of the papers have no significant difference. The sensitivity results show that the lowest measurable concentration is 75 mg/ml because in this concentration there are still appropriate color changes for the three reagents, whereas for concentrations less than 75 mg/ml the p-DAB reagent does not provide discoloration.

**Figure 5:**
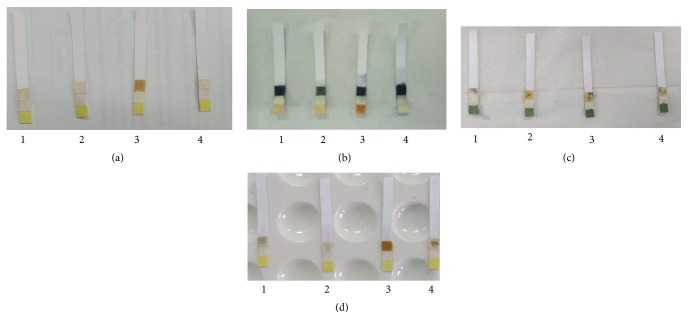
The selectivity of PAD in different papers: (1) Whatman No. 1, (2) Whatman No. 4, (3) Whatman No. 6, (4) Whatman 1 Chr. The PAD gives a different color on (a) reagent, (b) allopurinol, (c) paracetamol, and (d) caffeine.

The selectivity test was carried out to determine the selectivity of paper-based analytical device (PAD) in detecting allopurinol. The test was carried out by dipping PAD in a solution of caffeine and paracetamol. Both chemical drugs are usually contained in herbal medicine for gout.  Figure 5 shows that in PAD dipped in paracetamol, Folin reagent gave a green change, while the p-DAB reagent did not change color. In PAD dipped in caffeine, Folin reagent solution does not give a color change, while the p-DAB reagent gives a yellow color change. This result indicates that PAD is selective for allopurinol detection because it has a different color change when reacting with other interfering compounds.

### 3.4. Application of PAD for Real Sample Analysis

**Figure 6:**
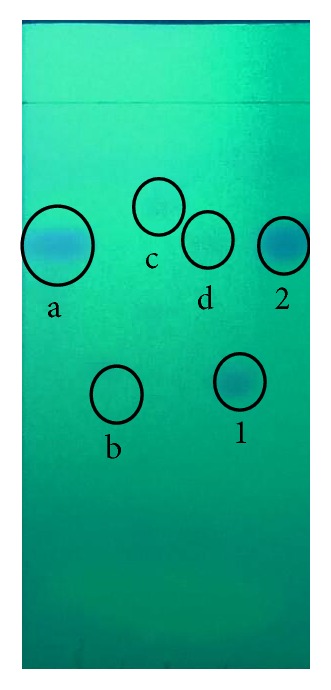
TLC analysis of herbal medicine samples: (a) sample a, (b) sample b, (c) sample c, (d) sample d; (1) allopurinol standard, (2) paracetamol standard.

To demonstrate performance of PAD for real sample, 4 herbal medicine samples were analyzed using TLC, Spectrophotometry UV-Visible, and PAD.  Figure 6 shows the TLC analysis of the sample. The TLC analysis shows that sample a and d are likely to contain paracetamol and sample b contains allopurinol because they have similar Rf to paracetamol and allopurinol standard, that is, 0.75 and 0.47, respectively. Sample c may contain another compound because it has different Rf value from both of them. The spot of sample b, c, and d is very weak, which might be due to the very small content of the compound in the sample.

Sample b that was predicted to contain allopurinol continued in the analysis using spectrophotometry UV.

To calculate the levels of allopurinol in sample b, a standard curve of allopurinol with concentrations of 6, 8, 10, 12, 14, and 16 *µ*g/ml was made. The six concentrations are measured at 252 nm wavelength. After that, a graph is drawn between the concentration and absorbance and the equation was obtained y = 0.0469x - 0.0357, with linear regression value (r2) of 0.9954. From the equation, the concentration of allopurinol in sample b was 7.44 *µ*g/ml.

For PAD detection, sample b was spiked with 70 mg/ml of allopurinol. The sample was dissolved in water and NaOH because herbal medicine is usually used by dissolving in water whereas allopurinol dissolves in NaOH. The result shows good agreement between the PAD method with TLC and spectrophotometry data. All of the methods confirm that sample b contains allopurinol.

## 4. Conclusion

Nine colorimetric reagents (Dragendorff reagent, ferric chloride, Folin-Ciocalteu reagent, sodium nitroprusside, p-DAB reagent, Schiff reagent, potassium chlorate, Tollens reagent, and sodium nitrite) were tested for allopurinol detection. From these reagents, Folin-Ciocalteu, p-DAB, and Tollens reagent can be applied to PAD. This PAD shows good sensitivity and selectivity. The developed PAD was also successful in detecting allopurinol in herbal medicine sample which also agrees with TLC and spectrophotometry data. This method is simple, rapid, instrument-free, and selective for on-site allopurinol analysis in herbal medicine.

## Data Availability

The data used to support the findings of this study are included in the article.
